# Exploring benzoxaborole derivatives as carbonic anhydrase inhibitors: a structural and computational analysis reveals their conformational variability as a tool to increase enzyme selectivity

**DOI:** 10.1080/14756366.2019.1653291

**Published:** 2019-08-18

**Authors:** Emma Langella, Vincenzo Alterio, Katia D’Ambrosio, Roberta Cadoni, Jean-Yves Winum, Claudiu T. Supuran, Simona Maria Monti, Giuseppina De Simone, Anna Di Fiore

**Affiliations:** a Istituto di Biostrutture e Bioimmagini, Consiglio Nazionale delle Ricerche, Naples, Italy;; b Institut des Biomolécules Max Mousseron (IBMM) UMR 5247 CNRS, ENSCM, Université de Montpellier, Ecole Nationale Supérieure de Chimie de Montpellier, Montpellier, France;; c Dipartimento di Chimica e Farmacia, Università Degli Studi di Sassari, Sassari, Italy;; d Neurofarba Department, Section of Pharmaceutical and Nutriceutical Sciences, Università Degli Studi di Firenze, Florence, Italy

**Keywords:** Carbonic anhydrase inhibitors, benzoxaborole derivatives, X-ray crystallography, binding free energy calculations, structure-based drug design

## Abstract

Recent studies identified the benzoxaborole moiety as a new zinc-binding group able to interact with carbonic anhydrase (CA) active site. Here, we report a structural analysis of benzoxaboroles containing urea/thiourea groups, showing that these molecules are very versatile since they can bind the enzyme assuming different binding conformations and coordination geometries of the catalytic zinc ion. In addition, theoretical calculations of binding free energy were performed highlighting the key role of specific residues for protein-inhibitor recognition. Overall, these data are very useful for the development of new inhibitors with higher selectivity and efficacy for various CAs.

## Introduction

Carbonic anhydrases (CAs) are metalloenzymes that catalyse the reversible hydration of carbon dioxide to bicarbonate and proton^1^^,^^2^. CAs are widespread in organisms belonging to all life kingdoms (i.e. bacteria, archaea, and eukarya) and evolved into eight distinct families, namely α, β, γ, δ, ζ, η, θ, and ι. α-CAs are present mainly in vertebrates, fungi, protozoa, corals, algae, in the cytoplasm of green plants, and in some bacteria^3^. β-CAs have been found in bacteria, algae, and chloroplasts of both monocotyledons and dicotyledons, as well as in many fungi and archaea^4^. γ-CAs have been reported in archaea, bacteria, and plants^5^, whereas δ- and ζ-CAs are found only in marine photosynthetic eukaryotes^6–8^. η- and θ-CAs have been discovered in *Plasmodium* species and in the marine diatom *Phaeodactylum tricornutum*, respectively^9^^,^^10^. Finally, the new ι-CA subclass was recently identified in the marine diatom *Thalassiosira pseudonana*^11^. All human (h) CAs belong to the α-class, with 12 catalytically active isoforms so far identified. These enzymes are extensively distributed in many tissues and organs, where they are involved in several essential physiological processes such as pH and CO_2_ homeostasis, respiration, transport of CO_2_/bicarbonate, electrolyte secretion, biosynthetic reactions, bone resorption, and calcification^2^^,^^12^. Consequently, their dysregulated expression level and/or abnormal enzymatic activity may be related to pathological conditions. For this reason, hCAs have been recognised as targets for the design of inhibitors or activators useful for biomedical applications^1^^,^^13–15^.

**Figure 1.**
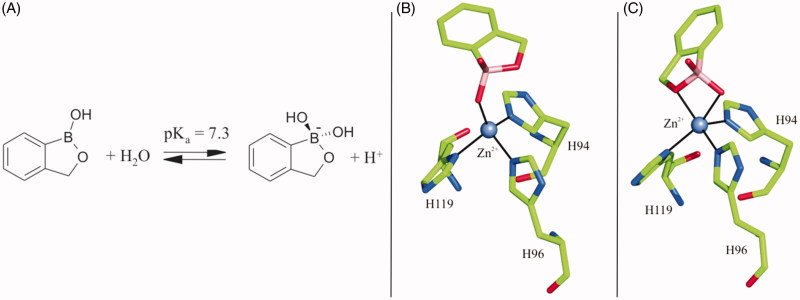
Schematic representation of benzoxaborole Lewis/Brønsted acidic properties (A). Binding of benzoxaborole to hCA II active site (PDB accession code 5JQ0)^24^ showing its tetrahedral (B) or trigonal bipyramidal coordination (C) to the catalytic zinc ion.

To date, several classes of CA inhibitors (CAIs) have been biochemically and structurally characterised, with sulphonamides and their isosteres (sulphamates and sulphamides) being the most studied^1^^,^^16^. However, very often the developed inhibitors were poorly selective, being able to inhibit indiscriminately all or most of the hCA isozymes^1^^,^^2^^,^^16^. With the aim to obtain more selective CAIs, recent years saw the continuous development and testing of molecules containing new chemotypes such as the carboxylates, polyamines, sulfocoumarins, and coumarins^17–21^. Among them the benzoxaborole derivatives were recently shown to have good inhibitory properties against α- and β-CAs from pathogenic fungi and protozoans^22^^,^^23^, as well to be effective on different hCA isoforms (Table 1)^24^. In particular, the lead compound benzoxaborole **1** was shown to act as a selective hCA I and hCA II inhibitor and to bind the enzyme active site in its tetrahedral anionic form independently from the pH used in crystallisation experiments (Figure 1). Interestingly, two different binding modes were observed: in the first one, one of the hydroxyl groups of the benzoxaborole was anchored to the catalytic zinc ion, completing its tetrahedral coordination (Figure 1(B)), whereas, in the second one, the inhibitor was bound to the zinc ion with two of its oxygen atoms, generating a trigonal bipyramidal coordination geometry of zinc (Figure 1(C))^24^. The substitution pattern at the benzoxaborole ring was deeply investigated too, showing that benzoxaboroles containing urea and thiourea moieties (compounds **2** and **3** in Table 1) were able to discriminate between the different CA isoforms^24^. Based on these interesting data, in this article, we further extend the characterisation of ureido/thioureido-benzoxaboroles by means of X-ray crystallography and binding free energy calculations.

**Table 1.** Inhibitor activity of benzoxaborole compounds against the hCA isoforms I, II, IX, and XII, as reported in the manuscript by Alterio et al.^24^

Inhibitor		*K_I_* (nM)			
		hCA I	hCA II	hCA IX	hCA XII
**1**		5690	8180	>50,000	>50,000
**2a**		654	730	1060	240
**2b**		557	439	925	184
**2c**		98	89	414	69
**3a**		548	1148	436	76
**3b**		380	1305	610	42

## Materials and methods

### X-ray crystallography

Crystals of homemade hCA II in complex with **2a**, **2b**, **3a**, and **3b** were prepared by the soaking technique^25^. In particular, hCA II native crystals were grown at room temperature using the vapour diffusion hanging drop method. Equal volumes of protein (10 mg/mL in 0.02 M Tris–HCl pH 8.0) and precipitant solution (1.3 M sodium citrate, 0.1 M Tris–HCl, pH 8.5) were mixed and equilibrated against 1 mL reservoir, containing the same precipitant solution. A few enzyme crystals were then transferred in a 2 μL drop of freshly prepared precipitant solution containing 40 mM inhibitor and 10% (v/v) glycerol as cryoprotectant agent. These crystals were kept in the soaking solution overnight for hCA II/**3a** and hCA II/**3b** adducts, whereas a very long soaking time was necessary for hCA II/**2a** and hCA II/**2b** (1 week for both cases). Crystals were frozen in a gaseous nitrogen stream prior to the diffraction experiment without further manipulations.

A complete X-ray data set was collected at 100 K by a copper rotating anode generator developed by Rigaku and equipped with a Rigaku Saturn CCD detector. Diffraction data were processed and scaled using program HKL2000 (HKL Research, Inc., Charlottesville, VA)^26^. Data collection statistics are reported in Table 2. The initial phases of hCA II/inhibitor structures were calculated using the atomic coordinates of the native enzyme with waters removed (PDB entry 1CA2)^27^. Electron density for all inhibitors was observed in the difference map after a single round of refinement. A model for each inhibitor was then built and introduced into the atomic coordinates for further refinement, which proceeded to convergence with alternating cycles of water addiction, manual rebuilding with the O program^28^, and energy minimisation and B-factor refinement with the CNS program^29^^,^^30^. Topology files for all compounds were obtained using the PRODRG server^31^. Refinement statistics for all hCA II/inhibitor adducts are summarised in Table 2. Coordinates and structure factors have been deposited in the Protein Data Bank (accession codes 6RVF, 6RVK, 6RVL, and 6RW1 for hCA II/**2a**, hCA II/**2b**, hCA II/**3a,** and hCA II/**3b**, respectively).

**Table 2.** X-ray diffraction data collection and refinement statistics.

	hCA II/**2a**	hCA II/**2b**	hCA II/**3a**	hCA II/**3b**
Cell parameter				
Space group	P2_1_	P2_1_	P2_1_	P2_1_
Unit cell parameters (Å, °)	*a* = 42.4	*a* = 42.4	*a* = 42.4	*a* = 42.4
	*b* = 41.4	*b* = 41.4	*b* = 41.5	*b* = 41.5
	*c* = 71.9	*c* = 72.2	*c* = 72.0	*c* = 72.1
	*β* = 104.2	*β* = 104.2	*β* = 104.5	*β* = 104.5
Data collection statistics				
Resolution limits (Å)	24.6–2.07	20.0–1.58	25.4–1.72	26.7–1.70
Temperature (K)	100	100	100	100
Total reflections	58731	154167	92543	117353
Unique reflections	14691	32939	24550	26109
Redundancy	4.0	4.7	3.8	4.5
Completeness (%)	98.0 (97.3)	98.3 (87.5)	94.3 (80.1)	96.5 (75.1)
R-merge*	0.125 (0.385)	0.062 (0.354)	0.092 (0.423)	0.062 (0.389)
Rmeas^§^	0.143 (0.464)	0.068 (0.429)	0.105 (0.538)	0.068 (0.493)
Rpim^¶^	0.068 (0.253)	0.028 (0.236)	0.050 (0.328)	0.028 (0.295)
<I>/<σ(I)>	10.0 (2.7)	20.8 (3.1)	11.6 (2.2)	19.4 (2.5)
Refinement statistics				
Resolution limits (Å)	24.6–2.07	20.0–1.58	25.4–1.72	26.7–1.70
R-work** (%)	18.4	16.9	17.6	18.1
R-free** (%)	22.9	19.7	20.9	20.7
r.m.s.d. from ideal geometry:				
Bond lengths (Å)	0.009	0.011	0.010	0.011
Bond angles (°)	1.4	1.7	1.6	1.5
Number of protein atoms	2039	2047	2051	2051
Number of inhibitor atoms	21	22	21	22
Number of water molecules	108	207	156	152
Average B factor (Å^2^)				
All atoms	13.96	14.44	12.77	15.57
Protein atoms	13.56	13.37	12.12	14.92
Inhibitor atoms	25.19	26.46	11.47	22.42
Water molecules	19.41	23.71	21.47	23.40
PDB accession code	6RVF	6RVK	6RVL	6RW1

*R-merge = ΣhklΣi|Ii(hkl)-<I(hkl)>|/ ΣhklΣiIi(hkl), where Ii(hkl) is the intensity of an observation and < I(hkl)> is the mean value for its unique reflection; summations are over all reflections.

§ Rmeas = Σ_hkl_{N(hkl)/[N(hkl)-1]}^1/2^Σ_i_|I_i_(hkl)-<I(hkl)>|/ Σ_hkl_Σ_i_I_i_(hkl).

¶ Rpim = Σ_hkl_{1/[N(hkl)-1]}^1/2^Σ_i_|I_i_(hkl)-<I(hkl)>|/ Σ_hkl_Σ_i_I_i_(hkl).

** Rfactor = Σ|Fo-Fc|/ΣFo. R-free is calculated as for R-work, but from data of the test set that was not used for refinement (Test Set Size = 7% for hCA II/**2a**, 3.2% for hCA II/**2b**, 4.5% for hCA II/**3a**, and 4.2% for hCA II/**3b**). Values in parentheses are referred to the highest resolution shell (2.11–2.07 Å for hCA II/**2a**, 1.61–1.58 Å for hCA II/**2b**, 1.75–1.72 Å for hCA II/**3a**, and 1.73–1.70 Å for hCA II/**3b**).

### Computational analysis

Theoretical calculations were carried out on the four crystallographic complexes of hCA II with **2a**, **2b**, **3a,** and **3b** as well as on two model complexes with 3a* and 3b*. The latter models were obtained by substituting the oxygen atom of urea moiety with a sulphur atom in the hCA II/**2a-b** crystal structure, using the Builder module of Insight II package (Insight2000, Accelrys, San Diego, CA). The partial atomic charges for ligands were obtained by quantum mechanical (QM) calculations (B3LYP/6–31G*) using the Gaussian16 software (Gaussian, Inc., Wallingford, CT)^32^ through the restrained electrostatic potential (RESP) fitting procedure as implemented in the PyRED server^33^^,^^34^. Since benzoxaborole derivatives bind to the catalytic metal ion in their tetrahedral anionic form^24^, the total charge for ligands was set at −1 e. According to our previous works^35^, a charge of +1.5 e was used for the zinc ion. The General AMBER^36^ and the AMBERff14SB force fields^37^ were used for the ligands and proteins, respectively^38^. Van der Waals parameters for the Zn^2+^ ion were adopted from the work of Li and Merz^39^ (*σ* = 1.271; *ɛ* (kcal/mol) = 0.00330286). Since boron atom is not parametrised in Amber-derived force fields, it was substituted with carbon, as reported in other modelling studies^40^^,^^41^, however, retaining the partial atomic charge of boron atom computed by PyRED as described above.

The binding free energies (Δ*G*bind in kcal/mol) for crystallographic complexes and the theoretical models were calculated using the molecular mechanics/generalised Born surface area (MM/GBSA) method^42^^,^^43^ implemented in AmberTools18^44^. Moreover, to identify the key protein residues responsible for the ligand-binding process, the binding free energy was decomposed on a *per-residue* basis.

According to MM/GBSA method, the binding free energy was estimated as follows:

(1) $$\Delta G_{\text{bind}}=G_{\text{complex}}-G_{\text{protein}}-G_{\text{ligand}}$$

where Δ*G*_bind_ is the binding free energy and *G*_complex_, *G*_protein_, and *G*_ligand_ are the free energies of complex, protein, and ligand, respectively. In details:

(2) $$\Delta G_{\text{bind}}=\Delta E_{\text{MM}}+\Delta G_{\text{sol}}-T\Delta S$$

(3) $$\Delta E_{\text{MM}}=\Delta E_{\text{elec}}+\Delta E_{\text{vdW}}$$

(4) $$\Delta G_{\text{sol}}=\Delta G_{\text{GB}}+\Delta G_{\text{SA}}$$

where Δ*G*_bind_ is the binding free energy in solution, Δ*E*_MM_ is the molecular mechanics energy, which comprises van der Waals (Δ*E*_vdW_) and electrostatic (Δ*E*elec) contributions; Δ*G*_sol_ is the solvation energy, and includes electrostatic (Δ*G*_GB_) and nonpolar (Δ*G*_SA_) interactions. The electrostatic solvation energy (Δ*G*_GB_) is evaluated using the Generalised Born method^45^, and the non-polar contribution is computed by the Linear Combination of Pairwise Overlaps (LCPO) method^46^. *T*Δ*S* is the change of conformational entropy on ligand binding, which is not included in our calculations. Indeed, for comparison of similar ligands, it is acceptable to exclude the entropy contribution in practise^43^^,^^47^.

## Results

To elucidate the binding mode of ureido/thioureido-benzoxaborole derivatives to the CA active site, we carried out a crystallographic study of the ubiquitous hCA II in complex with compounds **2a**, **2b**, **3a,** and **3b** (Table 1), characterised by the presence of a phenyl ring anchored to the urea/thiourea group through a linker of variable length. hCA II was selected as a model isoform for these studies since it is easy to crystallise and numerous data are available on adducts that it forms with inhibitors belonging to different classes^1^.

Crystals of hCA II adducts were obtained by soaking experiments following a protocol well described in literature^24^^,^^48^. In particular, native crystals of hCA II were grown using the hanging drop vapour diffusion technique at pH 8.5 and the obtained crystals were then transferred into a freshly prepared precipitant solution containing also the inhibitor. Data collection and refinement of all structures were performed as reported in the experimental section.

**Figure 2.**
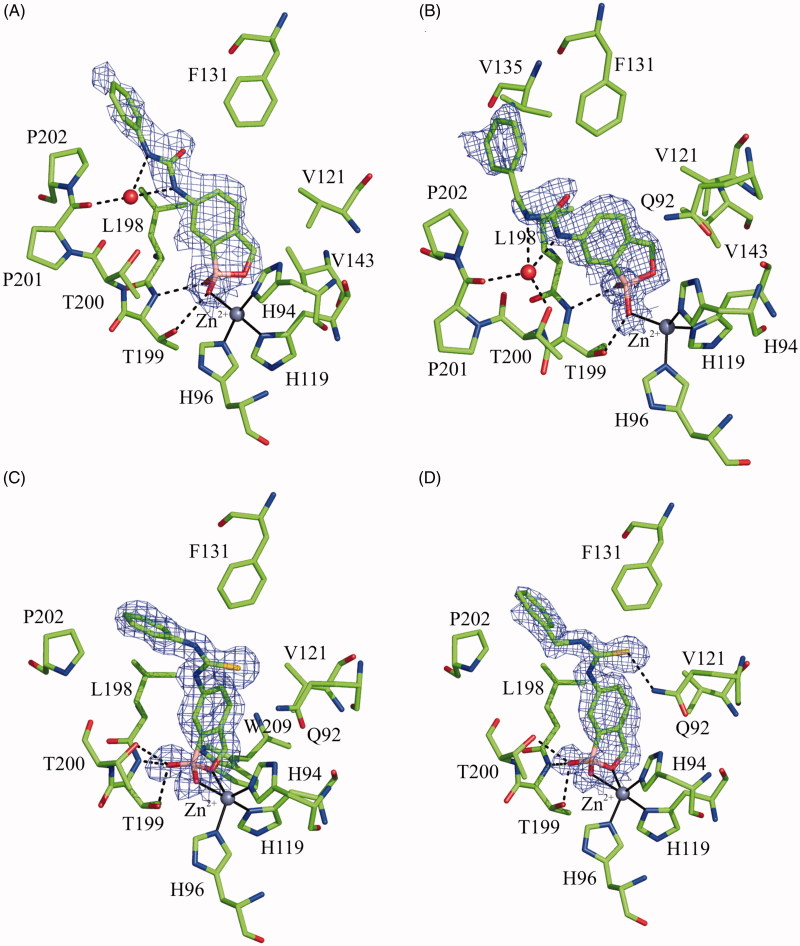
*σ*_A_-weighted |2Fo − Fc| map (contoured at 1.0 *σ*) relative to the inhibitor molecule in the hCA II adduct with **2a** (A), **2b** (B), **3a** (C), and **3b** (D). The zinc ion coordination and residues with a distance less than 4.0 Å from the inhibitor are also reported. Continuous lines show zinc ion coordination, whereas dashed lines indicate potential hydrogen bonds.

Analysis of the electron density maps since initial stages of the crystallographic refinement revealed that, as previously reported^24^, all benzoxaborole derivatives bind to the catalytic metal ion in their tetrahedral anionic form. Electron density maps were well defined for the whole inhibitor molecule in the case of hCA II/thiourea adducts, whereas a greater conformational variability was observed for the phenyl substituent of urea derivatives (Figure 2). The inhibitor binding did not alter hCA II three-dimensional structure. Indeed, the r.m.s.d. values calculated by superposition of all the Cα atoms of the hCA II/inhibitor adducts with those of the native protein were very low (r.m.s.d. values in the range of 0.18–0.21 Å).

**Figure 3.**
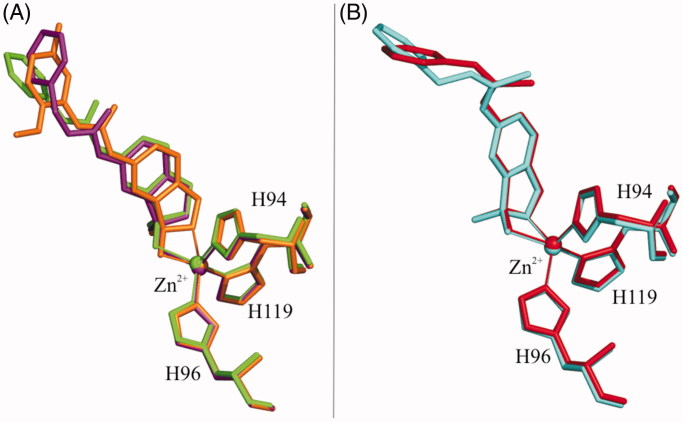
Structural superposition of benzoxaborole derivatives containing the urea (A) or thiourea (B) moiety. Compounds **2a**, **2b**, and **2c** are colored in green, purple and orange, respectively, whereas inhibitors **3a** and **3b** are in red and cyan. The zinc ion coordination is also reported.

A careful analysis of the hCA II/**2a** and hCA II/**2b** adduct structures showed that both inhibitors bind to the enzyme active site in a similar way, coordinating the catalytic zinc ion in tetrahedral geometry through one of the hydroxyl groups of the benzoxaborole moiety (Figure 2(A,B)), and being stabilised by many other polar and hydrophobic interactions. The conformations of the two inhibitors in the catalytic cavity are very similar (Figure 3(A)) with few differences related to the lengths of the phenyl-urea tails. The comparison of these structures with the previously reported compound **2c**^24^ showed that, despite a rather similar orientation of the benzoxaborole skeleton in the active site, the latter compound coordinates the zinc ion in a trigonal bipyramidal geometry (Figure 3(A)). Based on this finding and in agreement with previously reported data^24^, it is reasonable to assume that the two zinc coordination geometries, the tetrahedral and the trigonal bipyramidal, are in this case energetically equivalent and small changes in the orientation of the benzoxaborole skeleton can allow the transition from one to the other.

Superposition of compounds **3a** and **3b**, when bound to the enzyme active site, showed that, as observed for the urea-containing compounds, the binding modes of the two thiourea derivatives are similar to each other (Figure 3(B)). In this case, the two inhibitors bind the metal ion with two of their oxygen atoms, generating a trigonal bipyramidal coordination geometry. Additional polar and hydrophobic interactions contribute to stabilise the binding (Figures 2(C,D)).

**Figure 4.**
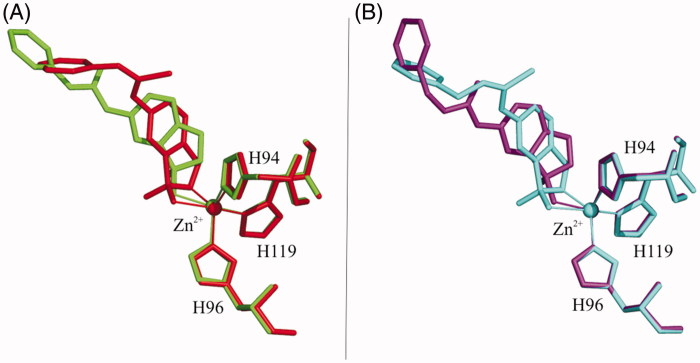
Superposition of ureido- and thioureido-benzoxaboroles. Inhibitors **2a** (green) and **3a** (red) containing the shorter linker are showed in panel A, whereas compounds **2b** (purple) and **3b** (cyan) in panel B.

**Figure 5.**
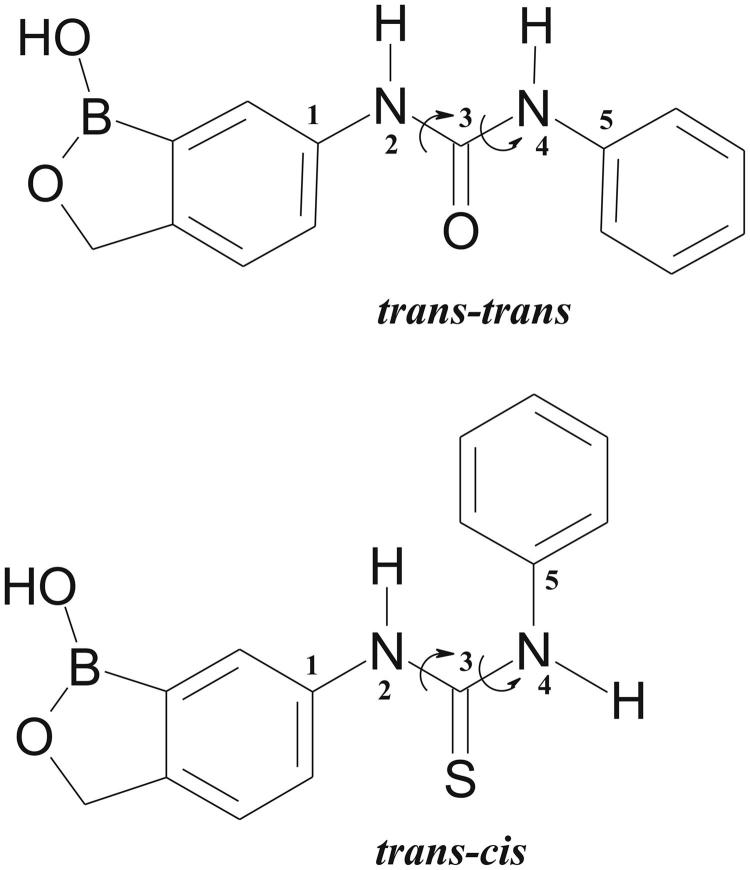
Schematic representation of *trans*-*trans* and *trans*-*cis* conformations of benzoxaboroles containing urea/thiourea groups.

In contrast, great differences are observed when the two groups of compounds are compared with each other. Indeed, even if compounds **2a** and **2b** differ from **3a** and **3b** only for one atom (an oxygen atom instead of a sulphur one) (see Table 1), their arrangement in the enzyme active site is completely different (Figure 4). One of the main differences is related to the geometry of the urea/thiourea moiety, which is *trans*-*trans* in the case of **2a** and **2b** and *trans*-*cis* in **3a** and **3b** (Figure 5). A question comes spontaneously to mind at this point: have urea and thiourea moieties an intrinsic preference for *trans-trans* and *trans-cis* geometry, respectively, that leads them to adopt a different binding orientation within the active site or are there specific protein/inhibitor interactions which influence this geometry and are responsible for their different binding mode to the enzyme?

To answer this question, a detailed analysis of urea and thiourea bis-phenyl derivatives contained in the Cambridge Structural Database (CSD)^49^ was carried out. This analysis revealed that these molecules do not have intrinsic conformational preferences, but they can assume both *trans-trans* and *trans-cis* conformations depending on the nature of the phenyl substituents and on the chemical environment (data not shown). Thus, the different binding geometries assumed by urea and thiourea derivatives within the hCA II active site are induced exclusively by specific interactions with the enzyme residues. Based on the inhibition data reported in Table 1, showing that ureido-benzoxaboroles have greater affinity for hCA II with respect to thiourea derivatives, it can be supposed that the binding conformation observed for compounds **2a** and **2b** is energetically more favoured with respect to that adopted by compounds **3a** and **3b**. Thus, to understand why thiourea-containing inhibitors do not assume the same conformation of urea-containing ones, binding free energy calculations were performed by using the MM/GBSA theoretical method^42^^,^^43^. This method allows decomposing the binding free energy on a per-residue basis in order to identify key protein residues responsible for the inhibitor binding mode. Calculations were carried out on the four crystallographic complexes as well as on two model complexes (hCA II/3a* and hCA II/3b*) obtained by substituting the oxygen atom of urea moiety with a sulphur atom in the hCA II/**2a** and hCA II**/2b** crystal structures. The latter two structures represent hypothetical models in which thiourea derivatives would adopt the same binding conformations observed for the corresponding urea derivatives.

Table 3 reports the protein residues giving a major contribution to the ligand-binding energy in the case of the crystallographic complexes, whereas Table 4 reports the same data for the model complexes. In the latter case, the energy differences with respect to the crystallographic structures are reported. The zinc ion contribution to ligand binding energy is not reported since, as reported in the literature, it is affected by the overestimation of the electrostatic interactions due to the high positive charge of Zn^2+^ ^50^.

**Table 3.** Per-residue decomposition of the binding free energy (kcal/mol) computed by the MM/GBSA method for the crystallographic hCA II adducts.

	hCA II/**2a**	hCA II/**2b**	hCA II/**3a**	hCA II/**3b**
Δ*G*_bind_-Gln92	−1.059	−1.555	−1.690	−3.772
Δ*E*_vdW_	−0.483	−0.601	−0.73	1.088
Δ*E*_elec_	−0.146	−1.358	0.314	−5.189
Δ*G*_GB_	−0.034	0.909	−0.959	1.004
Δ*G*_SA_	−0.396	−0.505	−0.315	−0.675
Δ*G*_bind_-Phe131	−1.224	−2.349	−2.295	−2.165
Δ*E*_vdW_	−0.379	−1.323	−1.445	−1.612
Δ*E*_elec_	−0.451	−0.338	−0.036	0.127
Δ*G*_GB_	0.496	0.555	0.387	0.487
Δ*G*_SA_	−0.89	−1.243	−1.201	−1.167
Δ*G*_bind_-Val135	−1.622	−1.500	−1.243	−1.483
Δ*E*_vdW_	−0.72	−0.66	−0.671	−0.783
Δ*E*_elec_	−0.331	−0.403	−0.058	−0.176
Δ*G*_GB_	0.078	0.179	0.009	0.117
Δ*G*_SA_	−0.650	−0.616	−0.523	−0.641
Δ*G*_bind_-Val143	−0.863	−1.493	−1.752	−1.614
Δ*E*_vdW_	0.276	−0.51	−0.786	−0.714
Δ*E*_elec_	−1.158	−1.024	−0.938	−0.903
Δ*G*_GB_	0.612	0.538	0.562	0.527
Δ*G*_SA_	−0.593	−0.497	−0.590	−0.524
Δ*G*_bind_-Leu198	−7.392	−7.211	−7.292	−7.062
Δ*E*_vdW_	−2.569	−2.34	−2.423	−2.496
Δ*E*_elec_	−3.666	−3.789	−3.864	−3.7
Δ*G*_GB_	0.752	0.794	0.775	0.876
Δ*G*_SA_	−1.909	−1.876	−1.780	−1.742
Δ*G*_bind_-Thr199	−3.374	−2.879	−3.350	−3.238
Δ*E*_vdW_	0.003	1.454	1.917	1.889
Δ*E*_elec_	−2.96	−3.466	−4.495	−4.243
Δ*G*_GB_	0.277	−0.128	0.018	−0.101
Δ*G*_SA_	−0.694	−0.739	−0.790	−0.783
Δ*G*_bind_-Thr200	−2.611	−1.883	−3.683	−3.616
Δ*E*_vdW_	−1.037	−0.092	−1.285	−1.212
Δ*E*_elec_	−1.618	−1.91	−1.343	−1.326
Δ*G*_GB_	0.917	1.048	0.018	0.05
Δ*G*_SA_	−0.873	−0.929	−1.073	−1.128
Δ*G*_bind_-Pro202	−2.775	−1.886	−1.647	−2.456
Δ*E*_vdW_	−1.484	−0.764	−0.704	−1.303
Δ*E*_elec_	−0.916	−0.408	−0.401	−0.654
Δ*G*_GB_	0.707	0.312	0.296	0.473
Δ*G*_SA_	−1.082	−1.026	−0.838	−0.972

Only residues contributing more than −1.0 kcal/mol to the binding are reported. Δ*E*_vdW_: van der Waals contribution; Δ*E*_elec_: electrostatic contribution; Δ*G*_GB_: generalised-Born solvation contribution; Δ*G*_SA_: non-polar solvation contribution.

**Table 4.** Binding free energy differences (kcal/mol) between hCA II/3a* and hCA II/3b* models and hCA II/**2a** and hCA II/**2b** crystallographic structures.

	Δ*a*^#^	Δ*b*^§^
Δ*G*_bind_-Gln92	−0.044	−0.135
Δ*G*_bind_-Phe131	15.054	1.486
Δ*G*_bind_-Val135	0.409	−0.075
Δ*G*_bind_-Val143	−0.001	0.005
Δ*G*_bind_-Leu198	−0.106	0.110
Δ*G*_bind_-Thr199	0.009	0.043
Δ*G*_bind_-Thr200	−0.050	−0.111
Δ*G*_bind_-Pro202	−0.080	−0.011

# hCA II/3a* - hCA II/**2a**.

§ hCA II/3b* - hCA II/**2b**.

For what concerns the crystallographic complexes, the protein residues mainly contributing to the binding are the same for all the ligands with quite comparable extents. In detail, Phe131, Val135, and Pro202 participate in the inhibitor binding through van der Waals (vdW) interactions (Table 3). These residues form a wide hydrophobic pocket at the mouth of the catalytic cleft, which is able to accommodate the phenyl ring of each ligand (Figure 2). Val143 and Leu198, located in the interior of the binding pocket, are involved in stabilising vdW and electrostatic interactions with the ligands, interacting with the benzoxaborole ring (Table 3). In addition, Thr199 and Thr200, at the bottom of the active site, establish electrostatic interactions with the inhibitors. Finally, Gln92, located at the entry of the active site, also contributes to inhibitor stabilisation. However, it is worth noting that a very strong polar interaction is observed in the case of hCA II/**3b** adduct, due to a hydrogen bond between the sulphur atom of thiourea moiety and Gln92 side chain, which is absent in the case of the other derivatives (Table 3 and Figure 2).

Regarding the model complexes (Table 4), the energy differences with respect to the crystal adducts are close to zero for all residues except for Phe131, which shows a destabilising energy contribution equal to 15.0 and 1.5 kcal/mol for derivatives 3a* and 3b*, respectively. These data indicate that thiourea derivatives would experience a significant destabilising interaction with Phe131 if they would adopt the same binding conformation of urea derivatives, likely due to an increase of the steric hindrance between their CS group and the Phe131 ring. These data suggest that the thiourea moiety turns towards a *trans*-*cis* conformation, to avoid this unfavourable interaction.

**Figure 6.**
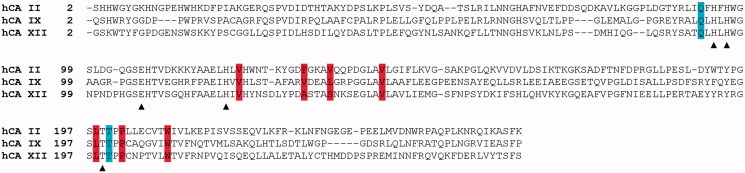
Structure-based sequence alignment of hCA II with the catalytic domain of tumor-associated hCAs IX and XII. Catalytic triad residues, Thr199 and Glu106 are indicated with a triangle, while hydrophobic and polar residues involved into protein-inhibitor binding are highlighted in red and cyan, respectively.

Our energetic calculations can help also to rationalise the interesting inhibition profile of the thiourea derivatives against the different hCA isoforms, with a particular focus on hCA IX and XII which have been recently recognised as valuable targets for cancer treatment and diagnosis^51^. Indeed, inhibition assays showed that these compounds inhibit with greater efficiency the tumour-associated hCA IX and XII with respect to the ubiquitous hCA II (Table 1). This can be explained based on the substitution of Phe131 with a valine and an alanine in hCA IX and XII (Figure 6), respectively. Indeed, these two smaller residues likely do not create the steric hindrance, originated by Phe131 in hCA II, for the positioning of the inhibitor CS group and permit an energetically more favoured arrangement of thiourea derivatives. These hypotheses are in agreement with previously reported structural studies^1^^,^^48^^,^^52^, which evidenced the important role of CA residue in position 131 in protein-inhibitor recognition and support the idea that region encompassing residues 131–135 constitutes a “hot zone”^53^ to be targeted for the design of novel CAIs with increased selectivity.

Altogether, studies reported here demonstrate that ureido- and thioureido-benzoxaborole derivatives are an interesting class of versatile CAIs. Indeed, these molecules can bind the enzyme active site assuming different binding conformations and coordination geometries depending on the interactions established with the enzyme active site residues. This feature can be efficiently used to design molecules with enhanced selectivity for pharmaceutically relevant hCA isoforms.

## References

[1] Alterio V, Di Fiore A, D’Ambrosio K, et al. Multiple binding modes of inhibitors to carbonic anhydrases: how to design specific drugs targeting 15 different isoforms? Chem Rev 2012;112:4421–68. 22607219 10.1021/cr200176r

[2] Supuran CT, De Simone G, eds. Carbonic anydrases as biocatalysts - from theory to medical and industrial applications. Amsterdam, Netherlands: Elsevier B.V; 2015.

[3] Di Fiore A, D’Ambrosio K, Ayoub J, et al, eds. Carbonic anhydrases - biochemistry and pharmacology of an evergreen pharmaceutical target. Amsterdam, Netherlands: Elsevier; 2019: 19–54.

[4] Rowlett RS Structure and catalytic mechanism of β-carbonic anhydrases. Subcell Biochem 2014;75:53–76. 24146374 10.1007/978-94-007-7359-2_4

[5] Ferry JG How to make a living by exhaling methane. Annu Rev Microbiol 2010;64:453–73. 20528692 10.1146/annurev.micro.112408.134051

[6] Tripp BC, Smith K, Ferry JG Carbonic anhydrase: new insights for an ancient enzyme. J Biol Chem 2001;276:48615–8. 11696553 10.1074/jbc.R100045200

[7] Lane TW, Saito MA, George GN, et al. Biochemistry: a cadmium enzyme from a marine diatom. Nature 2005;435:42. 15875011 10.1038/435042a

[8] Langella E, De Simone G, Esposito D, et al. z-Carbonic anhydrases In: Supuran CT, Nocentini A, eds. Carbonic anhydrases – biochemistry and pharmacology of an evergreen pharmaceutical target. Amsterdam, Netherlands: Elsevier; 2019:131–8.

[9] De Simone G, Di Fiore A, Capasso C, Supuran CT The zinc coordination pattern in the h-carbonic anhydrase from *Plasmodium falciparum* is different from all other carbonic anhydrase genetic families. Bioorg Med Chem Lett 2015;25:1385–9. 25765908 10.1016/j.bmcl.2015.02.046

[10] Kikutani S, Nakajima K, Nagasato C, et al. Thylakoid luminal q-carbonic anhydrase critical for growth and photosynthesis in the marine diatom Phaeodactylum tricornutum. Proc Natl Acad Sci USA 2016;113:9828–33. 27531955 10.1073/pnas.1603112113 PMC5024579

[11] Jensen EL, Clement R, Kosta A, et al. A new widespread subclass of carbonic anhydrase in marine phytoplankton. ISME J. 2019;13:2094–106. 31024153 10.1038/s41396-019-0426-8 PMC6776030

[12] Supuran CT Carbonic anhydrases: novel therapeutic applications for inhibitors and activators. Nat Rev Drug Discov 2008;7:168–81. 18167490 10.1038/nrd2467

[13] Supuran CT Applications of carbonic anhydrases inhibitors in renal and central nervous system diseases. Expert Opin Ther Pat 2018;28:713–21. 30175635 10.1080/13543776.2018.1519023

[14] Monti SM, Supuran CT, De Simone G Carbonic anhydrase IX as a target for designing novel anticancer drugs. Curr Med Chem 2012;19:821–30. 22214452 10.2174/092986712799034851

[15] Supuran CT, Di Fiore A, De Simone G Carbonic anhydrase inhibitors as emerging drugs for the treatment of obesity. Expert Opin Emerg Drugs 2008;13:383–92. 18537527 10.1517/14728214.13.2.383

[16] McKenna R, Supuran CT, Carbonic anhydrase inhibitors drug design In: McKenna R, Frost S, eds. Carbonic anhydrase: mechanism, regulation, links to disease, and industrial applications. Heidelberg, Germany: Springer Verlag; 2014:291–323.

[17] D’Ambrosio K, Carradori S, Monti SM, et al. Out of the active site binding pocket for carbonic anhydrase inhibitors. Chem Commun 2015;51:302–5. 10.1039/c4cc07320g 25407638

[18] Nocentini A, Carta F, Tanc M, et al. Deciphering the mechanism of human carbonic anhydrases inhibition with sulfocoumarins: computational and experimental studies. Chemistry 2018;24:7840–4. 29603439 10.1002/chem.201800941

[19] Scozzafava A, Supuran CT, Carta F Polyamines and a-carbonic anhydrases. Molecules 2016;21:1726. 10.3390/molecules21121726 PMC6273118 27983696

[20] Karioti A, Carta F, Supuran CT Phenols and polyphenols as carbonic anhydrase inhibitors. Molecules 2016;21:1649. 10.3390/molecules21121649 PMC6273245 27918439

[21] Lomelino CL, Supuran CT, McKenna R Non-classical inhibition of carbonic anhydrase. Int J Mol Sci 2016;17:1150. 10.3390/ijms17071150 PMC4964523 27438828

[22] Nocentini A, Supuran CT, Winum JY Benzoxaborole compounds for therapeutic uses: a patent review (2010- 2018). Expert Opin Ther Pat 2018;28:493–504. 29727210 10.1080/13543776.2018.1473379

[23] Nocentini A, Cadoni R, Dumy P, et al. Carbonic anhydrases from *Trypanosoma cruzi* and *Leishmania donovani* chagasi are inhibited by benzoxaboroles. J Enzyme Inhib Med Chem 2018;33:286–9. 29278948 10.1080/14756366.2017.1414808 PMC6009872

[24] Alterio V, Cadoni R, Esposito D, et al. Benzoxaborole as a new chemotype for carbonic anhydrase inhibition. Chem Commun 2016;52:11983–6. 10.1039/c6cc06399c 27722534

[25] Di Fiore A, Maresca A, Alterio V, et al. Carbonic anhydrase inhibitors: x-ray crystallographic studies for the binding of N-substituted benzenesulfonamides to human isoform II. Chem Commun 2011;47:11636–8. 10.1039/c1cc14575d 21952494

[26] Otwinowski Z, Minor W Processing of x-ray diffraction data collected in oscillation mode. Methods Enzymol 1997;276:307–26. 10.1016/S0076-6879(97)76066-X 27754618

[27] Eriksson AE, Jones TA, Liljas A Refined structure of human carbonic anhydrase II at 2.0 Å resolution. Proteins 1988;4:274–82. 3151019 10.1002/prot.340040406

[28] Jones TA, Zou JY, Cowan SW, Kjeldgaard M Improved methods for building protein models in electron density maps and the location of errors in these models. Acta Crystallogr A 1991;47:110–9. 2025413 10.1107/s0108767390010224

[29] Brunger AT, Adams PD, Clore GM, et al. Crystallography & NMR system: a new software suite for macromolecular structure determination. Acta Crystallogr D Biol Crystallogr 1998;54:905–21. 9757107 10.1107/s0907444998003254

[30] Brunger AT Version 1.2 of the crystallography and NMR system. Nat Protoc 2007;2:2728–33. 18007608 10.1038/nprot.2007.406

[31] Schuttelkopf AW, van Aalten DM PRODRG: a tool for high-throughput crystallography of protein-ligand complexes. Acta Crystallogr D Biol Crystallogr 2004;60:1355–63. 15272157 10.1107/S0907444904011679

[32] Frisch MJ, Trucks GW, Schlegel HB, et al. Gaussian 16 Rev. B.01. Wallingford, CT: 2016.

[33] Vanquelef E, Simon S, Marquant G, et al. R.E.D. Server: a web service for deriving RESP and ESP charges and building force field libraries for new molecules and molecular fragments. Nucleic Acids Res 2011;39:W511–7. 21609950 10.1093/nar/gkr288 PMC3125739

[34] Wang F, Becker JP, Cieplak P, Dupradeau FY R.E.D. python: object oriented programming for amber force fields. Paper presented at the 247^th^ American Chemical Society national meeting; 2014 March 16–20; Dallas, TX.

[35] De Simone G, Langella E, Esposito D, et al. Insights into the binding mode of sulphamates and sulphamides to hCA II: crystallographic studies and binding free energy calculations. J Enzyme Inhib Med Chem 2017;32:1002–11. 28738704 10.1080/14756366.2017.1349764 PMC6445192

[36] Wang J, Wolf RM, Caldwell JW, et al. Development and testing of a general amber force field. J Comput Chem 2004;25:1157–74. 15116359 10.1002/jcc.20035

[37] Maier JA, Martinez C, Kasavajhala K, et al. ff14SB: improving the accuracy of protein side chain and backbone parameters from ff99SB. J Chem Theory Comput 2015;11:3696–713. 26574453 10.1021/acs.jctc.5b00255 PMC4821407

[38] Langella E, D’Ambrosio K, D’Ascenzio M, et al. A combined crystallographic and theoretical study explains the capability of carboxylic acids to adopt multiple binding modes in the active site of carbonic anhydrases. Chemistry 2016;22:97–100. 26507456 10.1002/chem.201503748

[39] Li P, Merz KM. Jr., Taking into account the ion-induced dipole interaction in the nonbonded model of ions. J Chem Theory Comput 2014;10:289–97. 24659926 10.1021/ct400751u PMC3960013

[40] Tiwari R, Mahasenan K, Pavlovicz R, et al. Carborane clusters in computational drug design: a comparative docking evaluation using AutoDock, FlexX, Glide, and Surflex. J Chem Inf Model 2009;49:1581–9. 19449853 10.1021/ci900031y PMC2702476

[41] Johnsamuel J, Byun Y, Jones TP, et al. A convenient method for the computer-aided molecular design of carborane containing compounds. Bioorg Med Chem Lett 2003;13:3213–6. 12951095 10.1016/s0960-894x(03)00674-7

[42] Tsui V, Case DA Theory and applications of the generalized born solvation model in macromolecular simulations. Biopolymers 2000;56:275–91. 11754341 10.1002/1097-0282(2000)56:4<275::AID-BIP10024>3.0.CO;2-E

[43] Kollman PA, Massova I, Reyes C, et al. Calculating structures and free energies of complex molecules: combining molecular mechanics and continuum models. Acc Chem Res 2000;33:889–97. 11123888 10.1021/ar000033j

[44] Case DA, Ben-Shalom IY, Brozell SR, et al. AMBER 2018. San Francisco, CA: University of California; 2018.

[45] Onufriev A, Bashford D, Case DA Exploring protein native states and large-scale conformational changes with a modified generalized born model. Proteins 2004;55:383–94. 15048829 10.1002/prot.20033

[46] Weiser J, Shenkin PS, Still WC Approximate solvent-accessible surface areas from tetrahedrally directed neighbor densities. Biopolymers 1999;50:373–80. 10423546 10.1002/(SICI)1097-0282(19991005)50:4<373::AID-BIP3>3.0.CO;2-U

[47] Wang J, Morin P, Wang W, Kollman PA Use of MM-PBSA in reproducing the binding free energies to HIV-1 RT of TIBO derivatives and predicting the binding mode to HIV-1 RT of efavirenz by docking and MM-PBSA. J Am Chem Soc 2001;123:5221–30. 11457384 10.1021/ja003834q

[48] Bruno E, Buemi MR, Di Fiore A, et al. Probing molecular interactions between human Carbonic Anhydrases (hCAs) and a novel class of benzenesulfonamides. J Med Chem 2017;60:4316–26. 28453941 10.1021/acs.jmedchem.7b00264

[49] Groom CR, Bruno IJ, Lightfoot MP, Ward SC The Cambridge structural database. Acta Crystallogr B Struct Sci Cryst Eng Mater 2016;72:171–9. 10.1107/S2052520616003954 PMC4822653 27048719

[50] Hou T, Wang J, Li Y, Wang W Assessing the performance of the MM/PBSA and MM/GBSA methods. 1. The accuracy of binding free energy calculations based on molecular dynamics simulations. J Chem Inf Model 2011;51:69–82. 21117705 10.1021/ci100275a PMC3029230

[51] Monti SM, Supuran CT, De Simone G Anticancer carbonic anhydrase inhibitors: a patent review (2008–2013). Expert Opin Ther Pat 2013;23:737–49. 23672415 10.1517/13543776.2013.798648

[52] Menchise V, De Simone G, Alterio V, et al. Carbonic anhydrase inhibitors: stacking with Phe131 determines active site binding region of inhibitors as exemplified by the x-ray crystal structure of a membrane-impermeant antitumor sulfonamide complexed with isozyme II. J Med Chem 2005;48:5721–7. 16134940 10.1021/jm050333c

[53] Alterio V, Hilvo M, Di Fiore A, et al. Crystal structure of the catalytic domain of the tumor-associated human carbonic anhydrase IX. Proc Natl Acad Sci USA 2009;106:16233–8. 19805286 10.1073/pnas.0908301106 PMC2752527

